# Development of a prototype thermodynamic database for Nd-Fe-B permanent magnets

**DOI:** 10.1080/14686996.2021.1936627

**Published:** 2021-06-04

**Authors:** Taichi Abe, Masao Morishita, Ying Chen, Arkapol Saengdeejing, Kiyoshi Hashimoto, Yoshinao Kobayashi, Ikuo Ohnuma, Toshiyuki Koyama, Satoshi Hirosawa

**Affiliations:** a Elements Strategy Initiative Center for Magnetic Materials (ESICMM), National Institute for Materials Science, Tsukuba, Japan; b Department of Chemical Engineering and Materials Science, University of Hyogo, Hyogo, Japan; c School of Engineering, Tohoku University, Sendai, Japan; d Department of Material Science and Engineering, Tokyo Institute of Technology, Tokyo, Japan; e Department of Materials Design Innovation Engineering, Nagoya Univ., Nagoya, Japan

**Keywords:** Thermodynamic database, Gibbs energy, phase equilibria, metastable states, 40 Optical, magnetic and electronic device materials, 203 Magnetics / Spintronics / Superconductors, 605 Databases, data structure, ontology, 407 CALPHAD / Phase field methods

## Abstract

For the Nd-Fe-B permanent magnets, a prototype thermodynamic database of the 8-element system (Nd, Fe, B, Al, Co, Cu, Dy, Ga) was constructed based on literature data and assessed parameters in the present work. The magnetic excess Gibbs energy of the Nd_2_Fe_14_B compound was reassessed using thoroughly measured heat capacity data. The Dy-Nd binary system was reassessed based on formation energies estimated from *ab initio* calculations. The constructed database was applied successfully for estimations of phase equilibria during the grain boundary diffusion processes (GBDP) and the reactions in the hydrogenation decomposition desorption recombination (HDDR) processes.

## Graphical abstract

**Figure:**
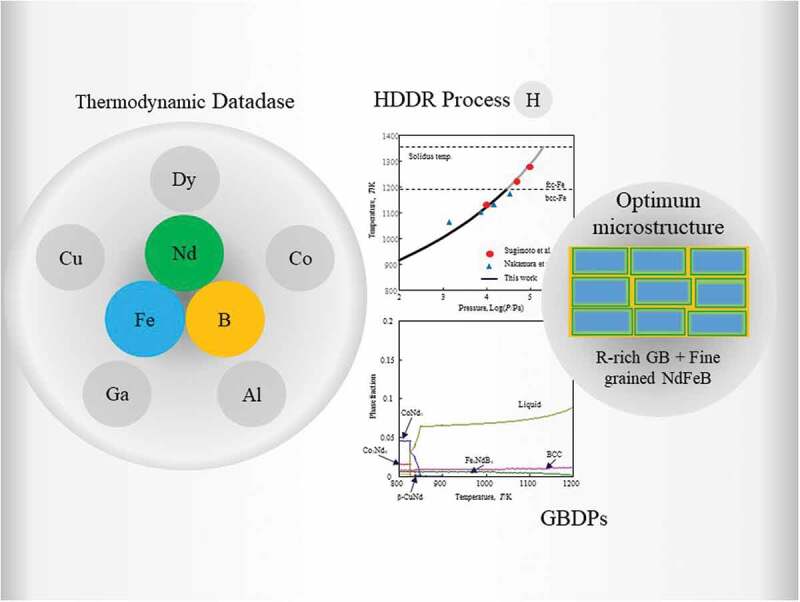

## 1. Introduction

**Figure 1.**
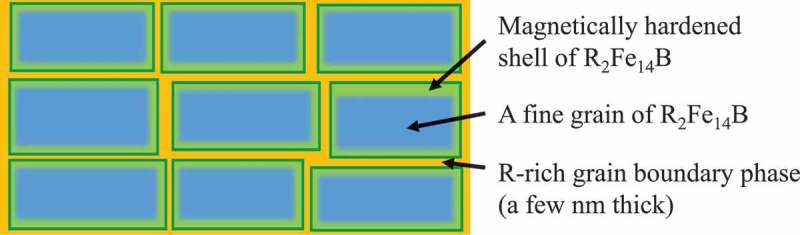
The proposed optimal microstructure of the Nd-Fe-B permanent magnets where the Nd_2_Fe_14_B phase (blue) is covered with a non-magnetic thin grain boundary phase (yellow)

Since Sagawa et al. reported the rare-earth permanent magnet in 1984 [1], which consists of the Nd_2_Fe_14_B compound as the main phase, it has been investigated intensively because of their high magnetization and magnetic anisotropy [2–7]. Nd-Fe-B based permanent magnets are widely used in electric vehicles, mobile phones, and electronic equipment that are essential to our daily lives. Fabrication of high-performance permanent magnets requires precise control of their microstructure. The optimum microstructure proposed in reference [8] is illustrated in Figure 1, where a Nd_2_Fe_14_B phase of micrometer-sized grains are coated by a thin non-magnetic grain boundary phase. Sasaki et al. [9,10] observed thoroughly the microstructure of Nd-based sintered magnets and revealed a variety of grain boundary phases coexisting with the main phase. These phase constitutions and morphology of the microstructures depend on the chemical compositions of the magnets and their sintering conditions. Thus, knowledge of thermodynamics and phase equilibria among the main and grain boundary phases is indispensable to improve the magnetic properties and further development of the Nd-Fe-B-based sintered magnets. Experimental accumulation of equilibrium phase diagrams of the Nd-Fe-B ternary and related systems is, however, limited. Matsuura et al. published the first phase diagram of the Nd-Fe-B system for B concentration lower than 55 at% in 1985 [11], which was followed by a few suggestions for minor changes [12,13]. Although this field has drawn intense interest, there has been less focus on the phase equilibria necessary to understand microstructural features and design the production processes. The empirical study became unsustainable in the industrial laboratories and has been left insufficient to describe the complex microstructures observed in the advanced products currently produced. The development of Nd-Fe-B permanent magnets has become dependent on know-how and experience accumulated in factories, which has limited the development of these materials.

There are three production routes to anisotropic bulk permanent magnets from Nd-Fe-B alloys; powder metallurgical sintering [1], rapid solidification followed by hot-deformation [14,15], and hydrogen-disproportionation-desorption-recombination (HDDR) [16]. Among these, the main route is powder metallurgical sintering, which uses a liquid phase above its eutectic temperature in a multicomponent system. The sintered body is then post-sinter annealed at lower temperatures close to and above the solidus temperature. Therefore, the phase equilibrium between the matrix phase Nd_2_Fe_14_B and the liquid phase has an important influence on the material structure. To prepare starting alloys for raw powder production, a rapid solidification process known as strip casting is used to avoid the formation of a large primary Fe phase [17]. Thus, the dynamic aspects of phase formation from the liquid phase [18] are also an important issue. In the second route, i.e., rapid solidification and hot-deformation, much finer grains of the main phase are produced by melt-spinning, and the alloys are hot-pressed and hot-deformed at ~1000 K by extrusion during which fine grains of the Nd_2_Fe_14_B phase align with the aid of the liquid phase. In the HDDR process, coarse powder of the Nd-Fe-B alloy is exposed to hydrogen gas at 990 K, causing it to disproportionate into nanosized grains of Fe, Fe_2_B, and NdH_2_, which recombine into submicrometer-sized Nd_2_Fe_14_B phase upon desorption of hydrogen. Thus, the system becomes Nd-Fe-B-H in the intermediate stage of the process. The equilibrium between a hydrogenated solid solution of the Nd_2_Fe_14_BH*_x_* phase and the disproportionated state is controlled by the addition of small amounts of additives such as Co as well as by the hydrogen pressure; thus, information on the phase equilibria of multicomponent systems would be useful for improving the HDDR processes. To fully understand and to develop precise microstructural engineering for these production processes, it requires information on the equilibria and dynamic aspects of the phase transformation, including thermodynamic data and the Gibbs energies of all relevant phases.

Commercial Nd-Fe-B magnets are not simply ternary Nd-Fe-B but are a multi-component system composed of, such as Nd, Fe, B, Dy, Cu, Ga, Al, and O [9,19] where Dy and micro-alloyed Cu and Ga are used to improve coercivity. A small amount of Al is also known to improve coercivity. Oxygen is an unavoidable impurity and the morphology of various oxide phases in the magnet is an important factor that affects on coercivity [20,21]. Existing empirical information is mostly limited up to ternary systems and is thus insufficient to describe the formation of the microstructures in the magnets. Moreover, technologies called ‘grain boundary diffusion’ [22] and ‘eutectic diffusion (or, grain boundary infiltration)’ [23] have recently been developed to modify the chemical compositions around grain boundaries by introducing extra elements to the surface of pre-densified pieces, which are either sintered or hot-pressed and deformed magnets, by means of grain boundary diffusion or liquid phase infiltration at relatively low temperatures. Thus, microstructural engineering is necessary to enhance coercivity without largely affecting the chemical composition of the main phase. In such processes, designing process parameters such as the processing temperature and compositions of both the base magnet and the source alloys for the diffusing elements requires understandings of local equilibria near the grain boundary.

The computational approach known as the combined *ab initio*/CALPHAD (CALculations of PHAse Diagrams) method [24,25] is a powerful tool for understanding obtained microstructures. We have previously constructed a database for the Nd-based system with oxygen and applied it to examine how oxygen behaves in the phase equilibria [26]. This database was limited to a 5-element system (Fe-Nd-B-Cu-O) because of difficulties to model the ionicity with oxygen using the ionic two sublattice model for an ionic liquid [27,28]. Considering the number of elements involved in commercial magnets our 5-element database is not suitable for estimating the phase equilibria of Nd-Fe-B permanent magnets. The primary difficulty in constructing a thermodynamic database for magnets is the limited thermodynamic data for systems containing lanthanide elements. In the first compilation of the binary phase diagrams [29] published in 1936, no binary systems with Nd were found. Even in the latest data book [30], most of them listed therein are partial diagrams based on the thermodynamic data measured before around the 1960s [31]. For ternary systems with lanthanide elements, available thermodynamic information is even more limited. The Nd_2_Fe_14_B compound has not been investigated well as discussed in section 3.2. Moreover, Nd-Fe-B based magnets contain various micro-alloying elements, such as Cu, Dy, Ga, and Al. The amounts of these elements are minute in overall compositions; however, these elements concentrate at grain boundaries and specific phases or form compounds during low-temperature heat treatments. Phase equilibria at grain boundaries are an important feature of these systems; however, the dilute solution approximation, which is usually applied for such elements, is inadequate for Nd-Fe-B based magnets. Therefore, here we construct a prototype thermodynamic database to cover a wider range of compositions and alloy systems with lanthanides. The new database currently includes 8 key elements (Fe, Nd, B, Dy, Co, Al, Cu, and Ga). Through the use of this database, we show that phase equilibrium calculations are an effective tool for understanding the actual processes of the Nd-Fe-B based permanent magnets.

## 2. Thermodynamic calculations

### 2.1 Gibbs energy of solution phase and intermetallic phases

The liquid, face centered cubic (fcc), body centered cubic (bcc), hexagonal close packed (hcp), and double hcp (dhcp) solution phases are modeled as substitutional solutions [24,25]. The molar Gibbs energy for a substitutional solution phase *Ø* is given as

(1) $$G_{m}^{\varphi }=\sum x_{i}^{\varphi }{\;}^{0}G_{i}^{\varphi }+RT\sum x_{i}^{\varphi }\ln x_{i}^{\varphi }+{\;}^{ex}G_{m}^{\varphi },$$

where ${\;}^{0}G_{i}^{\phi },{\;}^{ex}G_{m}^{\phi }$, $x_{i}^{\varphi }$ and *R* are the Gibbs energy for pure element *i* with a structure *Ø*, excess Gibbs energy, mole fraction of *i*, and gas constant, respectively. The Gibbs energies for the pure elements were obtained from the SGTE-Pure database version 5.0 [32,33]. The excess Gibbs energy is characterized by the use of the Redlich–Kister polynomial [34] as

(2) $${\;}^{ex}G_{m}^{\varphi }=x_{i}x_{j}\sum_{n=0}^{N}{\;}^{n}L_{i,j}^{\varphi }{\left(x_{i}-x_{j}\right)}^{n}.$$

The coefficients ${\;}^{n}L_{i,j}^{\phi }$ can be temperature dependent as ${\;}^{n}L_{i,j}^{\phi }={\;}^{n}A^{\phi }+{\;}^{n}B^{\phi }T$ where the coefficients ${\;}^{n}A^{\varphi }$ and ${\;}^{n}B^{\varphi }$ are fitted to experimental data.

Most of the intermetallic phases are treated as stoichiometric compounds in the present work. The Gibbs energy of a phase A*_p_*B*_q_* (*p* + *q* = 1) is only temperature dependent with respect to solid crystals of the pure elements at 298 K,

(3) $$G_{m}^{A_{p}B_{q}}=a+bT+p{\;}^{0}G_{A}^{\phi }+q{\;}^{0}G_{B}^{\phi },$$

where *a* and *b* are constants. For the Nd_2_Fe_14_B phase and non-stoichiometric compounds, sublattice models are adopted and are described later.

### 2.2 Magnetic excess Gibbs energy

In the CALPHAD-type thermodynamic assessments, the excess heat capacity from magnetic orderings under isobaric conditions is given by the Inden model [35,36]. Hillert and Jarl [37] applied a series of expansion to the logarithmic term in the Inden model. This model has been widely used in CALPHAD-type thermodynamic assessments. The isobaric excess heat capacities in the paramagnetic state, $C_{P}^{Para}$, and in the ferromagnetic states, $C_{P}^{Ferro}$, are given by

(4) $$\begin{array}{rl} & C_{p}^{Ferro}=2K_{\;}^{Ferro}R\left(\tau^{n}+\frac{\tau^{3n}}{3}+\frac{\tau^{5n}}{5}\right),\\ & C_{p}^{Para}=2K_{\;}^{Para}R\left(\tau^{-m}+\frac{\tau^{-3m}}{3}+\frac{\tau^{-5m}}{5}\right), \end{array}$$

where *K*^Ferro^ and *K*^Para^ are constants and *τ* is the normalized temperature defined by $\tau =T/T_{C}$where *T*_C_ is the Curie temperature. The values of *m* and *n* in the index are given empirically by Inden as 5 and 3, respectively. Using the Inden model the magnetic excess Gibbs energy, $G_{m}^{Mag}$, is given by

(5) $$G_{m}^{Mag}=RT\ln(\beta +1)g(\tau ),$$

where *β* is the thermodynamic magnetic moment and $g\left(\tau \right)$ is a temperature-dependent term. For *τ *< 1 it is given by

(6) $$g(\tau )=1-\frac{\frac{79}{140f}\tau^{-1}+\frac{474}{497}\left(\frac{1}{f}-1\right)\left(\frac{\tau^{3}}{6}+\frac{\tau^{9}}{135}+\frac{\tau^{15}}{600}\right)}{\frac{518}{1125}+\frac{11692}{15975}\left(\frac{1}{f}-1\right)}.$$

For *τ* > 1, it is

(7) $$g(\tau )=-\frac{\frac{\tau^{-5}}{10}+\frac{\tau^{-15}}{315}+\frac{\tau^{-25}}{1500}}{\frac{518}{1125}+\frac{11692}{15975}\left(\frac{1}{f}-1\right)}.$$

The value of *f* in these equations is a constant and given by the ratio of magnetic excess enthalpies as

(8) $$f\equiv \frac{\int_{T_{C}}^{\infty }C_{P}^{Para}dT}{\int_{0}^{T_{C}}C_{P}^{Ferro}dT}.$$

Inden [35] empirically determined *f* = 0.4 for a bcc phase and 0.28 for all of other phases.

### 2.3 Ab initio calculations

For the systems with lanthanide elements, experimental data such as phase equilibria and thermodynamic quantities are limited. To estimate the Gibbs energies of phases in the Dy-Nd system, we performed *ab initio* calculations. All calculations were based on density functional theory (DFT) that was implemented in the Vienna Ab initio Simulation Package (VASP) [38]. Projector-augmented wave (PAW) method is used to describe electron–ion interactions. The exchange and correlation are treated by the generalized gradient approximation (GGA) of Perdew–Burke–Ernzerhof (PBE). For the PAW potential of the rare-earth element, the *f* orbital is partially kept frozen in the core. Spin polarized calculations are included for all calculations. Further details of the present calculation can be found in references [39,40]. Cluster Expansion Method (CEM) [41] allows exploration of the ground state of random mixing in a certain lattice by expanding the internal energy of the system at a specific configuration, *E^n^*, as follows

(9) $$E^{n}=\sum_{\alpha }J_{\alpha }\sigma_{\alpha },$$

where *J_α_* is the effective cluster interaction (ECI) and *σ_α_* is the site occupation variable, defined based on the geometry of the structure of the system. For a set of ordered structures, the internal energies are calculated using *ab initio* calculations, and a set of ECIs can be extracted by matrix inversion. Furthermore, these ECIs can be used to estimate the energies of various configurations of the system using Eq. (9). Comparing the values of energies from direct band calculations and from the cluster expansion, an optimized set of ECIs can be obtained by fitting and can then be used in predicting the energies of a large number of other structures to explore the ground state of the system.

The formation energy of any given structure,$\Delta_{f}E^{\alpha }$, is calculated from the following equation

(10) $$\Delta_{f}E^{\alpha }=E_{0}^{\alpha }-\sum_{i}x_{i}E_{0}^{i},$$

where $E_{0}^{\alpha }$ is the energy of the structure *α* from *ab initio* calculations, *x_i_* is the mole fraction of element *i* presented in the structure, $E_{0}^{i}$ is the energy of element *i* in its reference structure. All energies are given for each atom. In this work, the Alloy Theoretic Automated Toolkit (ATAT) [42] is used to construct the cluster expansion model.

## 3. Construction of database for Nd-based magnets

### 3.1 Assessed binary and ternary subsystems

In the present work, a thermodynamic database for Nd-Fe-B magnets is constructed for the 8-element system, which includes: Al, B, Co, Cu, Dy, Fe, Ga, and Nd. Of 28 binary subsystems, 25 systems have been thermodynamically assessed based on the CALPHAD method [24,25,43]. Thermodynamic descriptions for the 25 assessed binary systems adopted in the present work are: Al-B [44], Al-Co [45], Al-Cu [46], Al-Dy [47], Al-Fe [48], Al-Ga [49], Al-Nd [50], B-Co [51], B-Cu [52], B-Dy [53], B-Fe [54], B-Ga [55], B-Nd [56], Co-Cu [57], Co-Dy [58], Co-Fe [59], Co-Ga [60], Co-Nd [61], Cu-Dy [62], Cu-Fe [63], Cu-Ga [64], Cu-Nd [65], Dy-Fe [66], Fe-Ga [67], and Fe-Nd [56]. The Dy-Ga [68–71] and Ga-Nd [71–75] binary systems have not yet been assessed because of a lack of experimental data. Although we set arbitrary parameters for ^0^*L* in Eq. (2) to reproduce liquidus lines in the Dy-Ga and Ga-Nd binary systems, they were not included in this database. Thus, full assessments of these binary systems are left for future works. The Dy-Nd binary system was reassessed in the present work and discussed in section 3.3. The results are summarized in Table 1.

**Table 1.** Assessed binary systems included in this database where ‘O’, and ‘×’ indicate assessed, and unassessed systems, respectively

	Al	B	Co	Cu	Dy	Fe	Ga
Nd	◯	◯	◯	◯	◯	◯	×
Ga	◯	◯	◯	◯	×	◯	
Fe	◯	◯	◯	◯	◯		
Dy	◯	◯	◯	◯			
Cu	◯	◯	◯				
Co	◯	◯					
B	◯						

This database includes 6 ternary systems taken from the literature, which are B-Fe-Nd [56], B-Co-Fe [76], Al-Cu-Dy [77], Al-Cu-Fe [78], Co-Cu-Fe [79], and Cu-Fe-Nd [80] ternaries. The Gibbs energy of the phases in these binary and some ternary systems can be downloaded from our database, Computational Phase Diagram Database (CPDDB) [81]. Descriptions of the phases included in the database are given in Table 2 where the phases with ‘*’ (B2-type, Dy_2_Fe_17_-type and Cu_5_Dy-type phases) are not yet unified because of the problem with sublattice configurations, which requires the Gibbs energy of metastable endmembers.

**Table 2.** Phase names, constituents and sublattices, structure types, and pearson symbols included in this database where ‘Va’ denotes vacancy and Phases with ‘*’ are not unified

Phase name	Constituents and Sublattices	Structure type	Pearson symbol
LIQUID	(Al,B,Co,Cu,Dy,Fe,Nd)_1_		
BCC_A2	(Al,B,Co,Cu,Dy,Fe,Nd)_1_(Va)_3_	W	cI2
FCC_A1	(Al,B,Co,Cu,Dy,Fe,Nd)_1_(Va)_1_	Cu	cF4
HCP_A3	(Al,B,Co,Cu,Dy,Fe,Nd)_1_(Va)_0.5_	Mg	hP2
DHCP	(Al,B,Co,Cu,Dy,Fe,Nd)_1_	Nd	hP4
RHOMB_B	(B,Cu)_1_	B	hR105
LAV_C14	(Al,Dy)_1_(Al,Dy)_2_	MgZn_2_	hP12
LAV_C15	(Al,Dy,Nd)_1_(Al,Co,Dy,Fe)_2_	MgCu_2_	cF24
LAV_C16	(Al,B,Cu)_1_(Al,Co,Fe)_2_	CuAl_2_	tI12
LAV_C36	(Al,Dy)_1_(Al,Dy)_2_	MgNi_2_	hP24
D011_1	(Co)_1_(Dy,Nd)_3_	Fe_3_C	oP16
D2_D_1*	(Co,Cu)_5_(Dy,Nd)_1_	CaCu_5_	hP6
C23	(Al)_1_(Dy,Nd)_2_	Co_2_Si	oP12
ALB12	(Al)_1_(B)_12_	···	tP52
ALB2	(Al)_1_(B)_2_	AlB_2_	hP3
AL5CO2	(Al)_0.714_(Co)_0.286_	Co_2_Al_5_	hP28
AL3CO	(Al)_0.75_(Co)_0.25_	···	···
AL13 CO4	(Al)_0.765_(Co)_0.235_	Co_4_Al1_3_	oP102
AL9CO2	(Al)_0.818_(Co)_0.182_	Co_2_Al_9_	mP22
ALCU	(Al,Cu)_1_(Cu)_1_	CuAl	mC20
AL2CU3	(Al)_2_(Cu)_3_	Al_2_Cu_3_	hP42
AL9CU11	(Al)_9_(Cu)_11_	Cu_4.25_(Cu_0.75_,A_l0.25_)_2_Al_4_	oI24
AL5CU8	(Al)_4_(Al,Cu)_1_(Cu)_8_	···	···
AL4CU9	(Al,Cu)_1_(Cu)_1_(Cu)_2_(Cu)_3_(Al,Cu)_6_	Cu_5_Al_8_	cI52
ALCU2	(Cu)_1_(Cu,Va)_1_(Al)_1_	AlCi_2_	hP6
AL2DY3	(Al)_2_(Dy)_3_	Gd_3_Al_2_	tP20
ALDY	(Al)_1_(Dy)_1_	DyAl	oP16
AL3DY	(Al)_3_(Dy)_1_	HoAl_3_	hR60
D024	(Al)_3_(Dy)_1_	TiNi_3_	hP16
AL2DY	(Al,Cu,Dy)_2_(Al,Cu,Dy)_1_	MgCu_2_	cF24
AL2FE	(Al)_2_(Fe)_1_	Al_2_Fe	aP18
AL13FE4	(Al)_0.6275_(Fe)_0.235_(Al,Va)_0.1375_	Fe_4_Al_13_	mC102
AL5FE2	(Al)_5_(Fe)_2_	FeAl_2.8_	oC24
AL5FE4	(Al,Fe)_1_	Cu_5_Zn_8_	cI52
AL4ND	(Al)_0.8_(Nd)_0.2_	BaAl_4_	tI10
AL11ND3	(Al)_0.7857_(Nd)_0.2143_	La_3_Al_11_	oI28
AL3ND_B	(Al)_0.75_(Nd)_0.25_	···	hP12
AL3ND_A	(Al)_0.75_(Nd)_0.25_	Mg_3_Cd	hP8
ALND	(Al)_0.5_(Nd)_0.5_	DyAl	oP16
ALND3	(Al)_0.25_(Nd)_0.75_	Mg_3_Cd	hP8
BCO	(B)_0.5_(Co)_0.5_	FeB	oP8
CO3B	(B)_0.25_(Co)_0.75_	Fe_3_C	oP16
B2DY	(B)_2_(Dy)_1_	AlB_2_	hP3
B4DY	(B)_4_(Dy)_1_	UB_4_	tP20
B6DY	(B)_6_(Dy)_1_	CaB_6_	cP7
B12DY	(B)_12_(Dy)_1_	UB_12_	cF52
B66DY	(B)_66_(Dy)_1_	YB_66_	cF1936
FEB	(FE)_0.5_(B)_0.5_	FeB	oP8
ND2B5	(B)_0.7143_(Nd)_0.2857_	Pr_2_B_5_	mS56
NDB4	(B)_0.8_(Nd)_0.2_	UB_4_	tP20
NDB6	(B)_0.8571_(Nd)_0.1429_	CaB_6_	cP7
NDB66	(B)_0.9851_(Nd)_0.0149_	YB_66_	cF1936
CO17DY2	(Co)_15_(Co_2_,Dy)_1_(Co_2_,Dy)_2_	Zn_17_Th_2_	hR57
CO7DY12	(Co)_7_(Dy)_12_	Ho_12_Co_7_	mP38
CO3DY1	(Co)_3_(Dy)_1_	PuNi_3_	hR36
CO7DY2	(Co)_7_(Dy)_2_	Gd_2_Co_7_	hR54
CO17ND2*	(Co)_17_(Nd)_2_	Zn_17_Th_2_	hR57
CO19ND5	(Co)_19_(Nd)_5_	Co_19_Ce_5_	hR24
CO7ND2	(Co)_7_(Nd)_2_	Ce_2_Ni_7_	hP36
CO3ND	(Co)_3_(Nd)_1_	PuNi_3_	hR36
CO3ND2_A	(Co)_3_(Nd)_2_	La_2_Ni_3_	oS20
CO3ND2_B	(Co)_3_(Nd)_2_	···	···
CO3ND4	(Co)_3_(Nd)_4_	···	···
CO2ND5	(Co)_2_(Nd)_5_	Mn_5_C_2_	mS28
CUDY*	(Cu)_0.5_(Dy)_0.5_	CsCl	cP2
CU7DY	(Cu)_0.875_(Dy)_0.125_	···	···
CU5DY_L	(Cu)_0.833333_(Dy)_0.166667_	Be_5_Au	cF24
CU9DY2	(Cu)_0.8182_(Dy)_0.1818_	···	···
CU7DY2	(Cu)_0.7778_(Dy)_0.2222_	···	···
CU2DY	(Cu)_0.6667_(Dy)_0.3333_	KHg_2_	oI12
CU6ND	(Cu)_0.8571_(Nd)_0.1429_	Cu_6_Ce	oP28
CU4ND	(Cu)_0.8_(Nd)_0.2_	···	···
CU7ND2	(Cu)_0.7778_(Nd)_0.2222_	···	···
CU2ND	(Cu)_0.6667_(Nd)_0.3333_	KHg_2_	oI12
CUND_B	(Cu)_0.5_(Nd)_0.5_	···	···
CUND_A	(Cu)_0.5_(Nd)_0.5_	FeB	oP8
DYFE3	(Dy)_0.25_(Fe)_0.75_	PuNi_3_	hR36
B2_ALCO*	(Al,Co)_0.25_(Al,Co)_0.25_(Al,Co)_0.25_(Al,Co)_0.25_	CsCl	cP2
DY6FE23	(Dy)_0.206897_(Fe)_0.793103_	Th_6_Mn_23_	cF116
DY2FE17*	(Dy)_0.105263_(Fe)_0.894737_	Th_2_Ni_17_	hP38
ND2FE14B	(B)_0.0588_(Fe)_0.8236_(Dy,Nd)_0.0588_(Dy,Nd)_0.0588_	Nd_2_Fe_14_B	tP68
FE4NDB4	(B)_0.4391_(Fe)_0.4391_(Nd)_0.1218_	···	···
FE2ND5B6	(B)_0.4615_(Fe)_0.1539_(Nd)_0.3846_	Pr_5_Co_2_B_6_	hR39
FE13ND6CU	(Cu)_0.05_(Fe)_0.65_(Nd)_0.30_	Pr_6_Co_13_Ga	tI80
TAU1	(Al)_0.615385_(Cu)_0.307692_(Dy)_0.076923_	Mn_12_Th	t126
TAU2*	(Al,Cu)_0.894737_(Dy)_0.105263_	Th_2_Zn_17_	hR57
TAU3*	(Al,Cu)_0.833333_(Dy)_0.166667_	CaCu_5_	hP6
TAU4	(Al)_0.6_(Cu)_0.2_(Dy)_0.2_	Al_4_Ba	oI10
TAU5	(Al)_0.578948_(Cu)_0.2210,526_(Dy)_0.210526_	···	···
TAU6	(Al,Cu)_0.75_(Dy)_0.25_	Ni_3_Pu	hR36
TAU7	(Al)_0.333333_(Cu)_0.333333_(Dy)_0.333334_	AlNiZr	hP9
ALCUFE_T	(Fe)_0.125_(Al,Cu)_0.255_(Al)_0.620_	···	···
ALCUFE_W	(Fe)_1_(Cu)_2_(Al)_7_	Cu_2_FeAl_7_	tP40
ALCUFE_P	(Fe)_1_(Al,Cu)_10_(Al)_10_	···	oF116
NDH2	(Nd)_1_(H)_2_	CaF_2_	cF12
GAS	(B,B_10_H_14_,B_1_H_1_,B_1_H_2_,B_1_H_3_,B_2_,B_2_H_6_,B_5_H_9_,		
	Fe,Fe_1_H_1_,Fe_2_,H,H_2_,Nd)_1_		

### 3.2 Gibbs energy of the Nd_2_Fe_14_B phase

**Figure 2.**
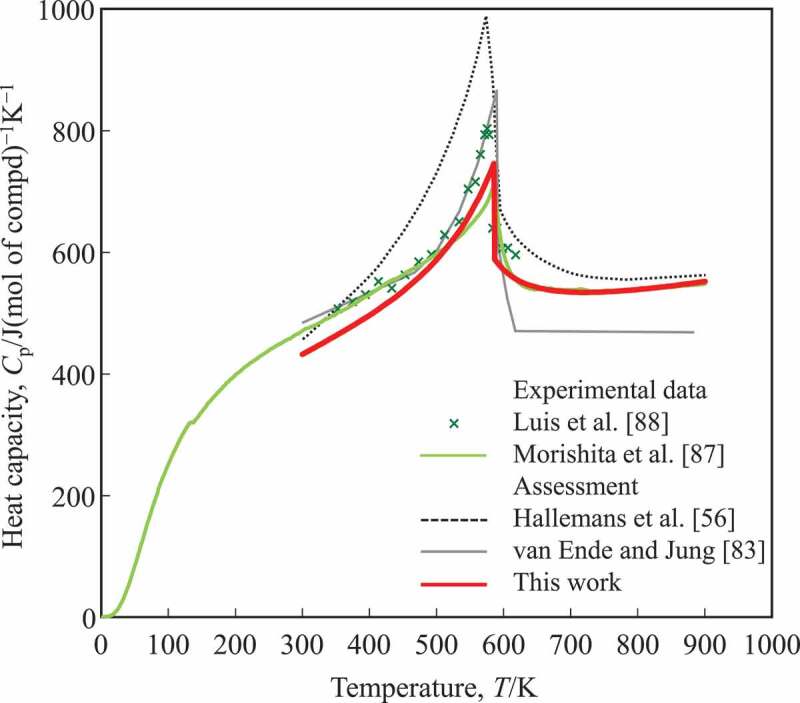
Heat capacity of the Nd_2_Fe_14_B phase calculated in this work, compared to previous data: Luis et al. [88], Morishita et al. [87], Hallemans et al. [56], and van Ende and Jung [83]. In the present work, Curie temperature is *T*_c_ = 585.3 K and thermodynamic magnetic moment is *μ*_B_ = 0.8

A CALPHAD-type assessment of the Nd-Fe-B ternary system was first performed by Hallemans et al. [56] in 1995. Because of its importance, the Nd-Fe-B ternary system has been reassessed and modified several times based on the Hallemans’ results. Van Ende et al. [82,83] have applied a quasi-chemical model for the liquid to describe the short-range ordering in liquid. Zhou et al. [84] have considered some metastable compounds. More recently Chen et al. [85] reassessed the B-Nd binary system. One of the major problems pointed out in these assessments is the uncertainty of the phase equilibria between liquid and borides in the B-Nd binary system assessed by Hallemans et al. [56]. Recently, Hanindriyo et al. [86] reconsidered the stability of borides in the B-Nd system on the bases of theoretical calculations. For expansion to higher-order systems, and to improve the descriptions of the Gibbs energy, the Gibbs energy functions adopted in the present work are based on the assessment of Hallemans et al. In the previous assessments [56,82–86], there is one significant uncertainty, which is the Gibbs energy of the Nd_2_Fe_14_B phase because the heat capacity of the compound is not well characterized at high temperatures where the magnetic transition occurs. To improve on this uncertainty, Morishita et al. [87] measured the heat capacity of Nd_2_Fe_14_B. According to their experimental results, it has a *T*_C_ and the magnetic moment are 585.3 K and 2.04, respectively. The *f* parameter in Eq. (8) is 0.24. Thus, we adopted *T*_C_ and *f* values from their measurements. Since the peak on the specific heat due to the magnetic transition becomes too high with 2.4 in Hallemans et al. or 2.04 in Morishita et al., in the present work the thermodynamic magnetic moment, *β*, in Inden model was reassessed and obtained a value of 0.8 from parameter fitting to the specific heat [87]. For the non-magnetic part of the heat capacity, Kopp-Neuman rule [43] was applied. A comparison between the measured [87,88] and calculated [56,83] heat capacities is presented in Figure 2. This refinement on the magnetic transition did not affect phase equilibria [56] from low to the elevated temperatures.

As Dy is one of essential elements for Nd-Fe-B permanent magnets, it is necessary to consider Dy in the Gibbs energy expression of the Nd_2_Fe_14_B phase in the database. Thus, the Nd_2_Fe_14_B phase is described by a four sublattice model defined by (Dy, Nd)_1_(Dy, Nd)_1_(Fe)_14_(B)_1_, which is based on the Wyckoff positions (4 *f* and 4 *g*) of lanthanide elements in the compound. In this description, Dy and Nd can mix in the first and second sublattices. The third and the fourth sublattices are occupied by only Fe and B atoms, respectively. The Gibbs energy of the phase for one mole of atoms is thus given by

(11) $$\begin{array}{rl} & {\text{\textbackslash{}G}}_{m}^{Nd_{2}Fe_{14}B}=y_{Dy}^{\left(1\right)}y_{Dy}^{\left(2\right)}G_{m}^{Dy:Dy:Fe:B}+y_{Dy}^{\left(1\right)}y_{Nd}^{\left(2\right)}G_{m}^{Dy:Nd:Fe:B}\\ & \;\;\;\;\;\;\;\;\;\;\;\;\;\;\;\;\;\;\;\;\;\;+y_{Nd}^{\left(1\right)}y_{Dy}^{\left(2\right)}G_{m}^{Nd:Dy:Fe:B}+y_{Nd}^{\left(1\right)}y_{Nd}^{\left(2\right)}G_{m}^{Nd:Nd:Fe:B}\;\;\;\\ & \;\;\;\;\;\;\;\;\;\;\;\;\;\;\;\;\;\;\;\;\;\;\;+\frac{1}{17}RT\left(y_{i}^{\left(1\right)}\ln y_{i}^{\left(1\right)}+y_{i}^{\left(2\right)}\ln y_{i}^{\left(2\right)}\right)\\ & \;\;\;\;\;\;\;\;\;\;\;\;\;\;\;\;\;\;\;\;\;\;\;+y_{Dy}^{\left(1\right)}y_{Nd}^{\left(1\right)}L_{\;}^{Dy,Nd:*:Fe:B}+y_{Dy}^{\left(2\right)}y_{Nd}^{\left(2\right)}L_{\;}^{*:Dy,Nd:Fe:B}, \end{array}$$

where $y_{i}^{\left(n\right)}$ is a mole fraction of element *i* on the *n*-th sublattice. $G_{m}^{Dy:Dy:Fe:B}$, $G_{m}^{Dy:Nd:Fe:B}$, $G_{m}^{Nd:Dy:Fe:B}$, and $G_{m}^{Nd:Nd:Fe:B}$ are Gibbs energies of the end-members of (Dy)_1_(Dy)_1_Fe_14_B_1_, (Dy)_1_(Nd)_1_Fe_14_B_1_, (Nd)_1_(Dy)_1_Fe_14_B_1_, (Nd)_1_(Nd)_1_Fe_14_B_1_, respectively. $L_{\;}^{Dy,Nd:*:Fe:B}$ and $L_{\;}^{*:Dy,Nd:Fe:B}$ are the regular mixing parameters of the first and second sublattices where ‘*’ denotes that it does not depend on the species on that sublattice. The Gibbs energy of (Dy)_1_(Dy)_1_Fe_14_B_1_ is estimated from the previous assessment [83] and is slightly more negative than (Nd)_1_(Nd)_1_Fe_14_B_1_. Recently, Saito et al. [89] measured a preferential site of Dy in the Nd_2_Fe_14_B structure by means of neutron diffraction and, estimated the *G* and *L* parameters in Eq. (11) from *ab initio* calculations. In this database, we adopted their assessment listed in Table 2 of reference [89] except for the (Nd)_1_(Nd)_1_Fe_14_B_1_ and (Dy)_1_(Dy)_1_Fe_14_B_1_. These parameters adequately explain the experimentally measured partitioning behaviors of Dy and Nd [89]. Although weak miscibility gaps at low temperatures (around 150 K) are expected, these behaviors are likely difficult to confirm experimentally.

### 3.3 Combined ab initio/CALPHAD approach for the Dy-Nd system

Most inter-lanthanide systems have a large solubility and a wide single-phase region, and the Dy-Nd binary system is a typical example of this type of diagram [31]. The first Dy-Nd binary diagram based on experimental data [31,90–92] was proposed by Gschneidner and Calderwood in 1982 [93,94]. This diagram was based on experimental data by Kobzenko et al. [92], which consisted of Liquid, bcc, hcp, dhcp, and δ(α-Sm-type structure) solid solution phases. Because of the peritectoid reaction, dhcp+hcp↔α-Sm, was not confirmed by other researchers. As Gschneidner and Calderwood discussed in the literature [94], we concluded that the formation of δ phase is a martensitic-type and has a congruent point. They also pointed out that the evidence for this type of phase transformation is in the Nd-Sc binary system. Consequently, a continuous solid solution forms between the hcp and dhcp phases regardless of the fact that invariant reactions between the phases are thermodynamically required. Therefore, it is clear that more experimental and theoretical information is required to confirm phase equilibria in inter-rare-earth systems. A CALPHAD-type assessment of this binary system was performed by van Ende and Jung [83] where the δ phase modeled as an intermetallic phase has a congruent point and a phase transition between hcp and dhcp was not considered. According to their assessment, both Dy and Nd have an hcp structure.

**Figure 3.**
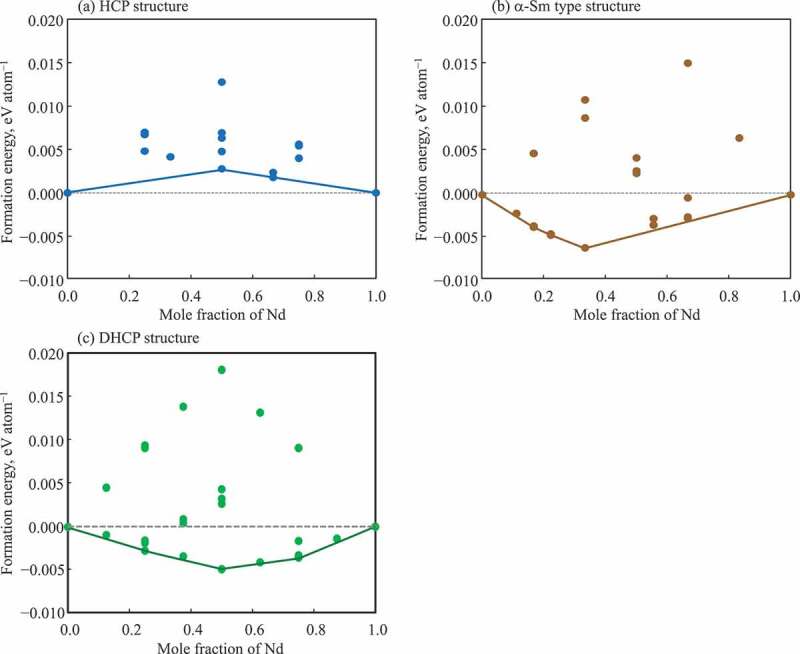
Calculated mixing energies of various ordered structures in (a) hcp lattice, (b) α-Sm-type lattice, and (c) dhcp lattice. Solid line indicates convex hull, which is close to the ideal where the formation energy is zero in these structures

**Figure 4.**
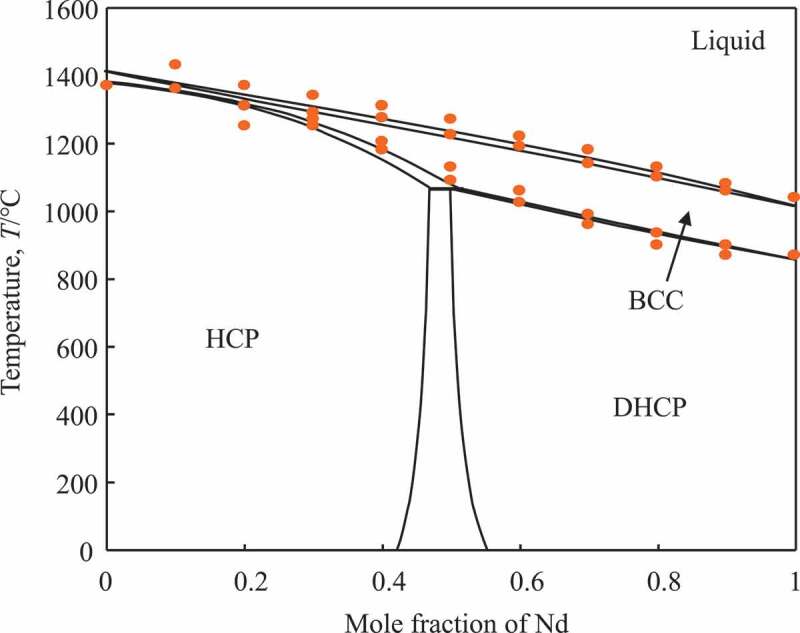
Calculated Dy-Nd binary phase diagram. Open symbols are experimental data [92]

In this work, we estimated the mixing enthalpies in the hcp, α-Sm, and dhcp structures from *ab initio* calculations as presented in Figure 3(a,b,c). Using phase boundary data [92] and the estimated interaction parameters for the hcp and dhcp phases, the Dy-Nd phase diagram was assessed in this work. The obtained parameters are ${\;}^{0}L_{Dy,Nd}^{Liquid}$=0, ${\;}^{0}L_{Dy,Nd}^{BCC}$=0, ${\;}^{0}L_{Dy,Nd}^{HCP}$=−1.6, and ${\;}^{0}L_{Dy,Nd}^{DHCP}$=−0.7 kJ/mol for Liquid, bcc, hcp, and dhcp phases, respectively. With these parameters, the phase boundary data and the mixing enthalpies can be reproduced well. As these values are close to zero, the behaviors of these solution phases are close to ideal. It suggests that the formation of intermediate compounds may not be plausible in this system. The obtained phase diagram is presented in Figure 4 with experimental data [92]. The small negative mixing energy, ${\;}^{0}L_{Dy,Nd}^{\alpha -Sm}$=−1.5 kJ/mol, for the α-Sm, may suggest that the α-Sm type solid solution or a DyNd compound were not stable in this system. To reproduce the experimental data (open symbols) in Figure 4, a peritectoid reaction was introduced over a narrow composition range at Dy-50at%Nd in this assessment. To refine the phase diagram, the stability of δ phase and the invariant reaction need to be examined experimentally.

## 4. Application of the database

### 4.1 Grain boundary diffusion process

Grain boundary diffusion process (GBDP) is one of the effective methods to fabricate high-performance magnets and to reduce the total amount of heavy rare-earth elements, such as Dy, used in magnets. In this section, the alloy Nd_10.9_Pr_3.3_Dy_0.2_Fe_77.5_Co_2.4_B_5.7_Ga_0.1_Cu_0.1_ (at%) is examined to demonstrate how the constructed database can be applied to estimate microstructural changes of the alloy. The thermal history was taken from reference [95] where Dy was deposited on the surface and diffused into the specimen at 1173 K followed by annealing at 823 K for several hours. For this GBDP, thermodynamic calculations using the present database can provide insights into changes of the phase constitutions and phase fractions.

**Figure 5.**
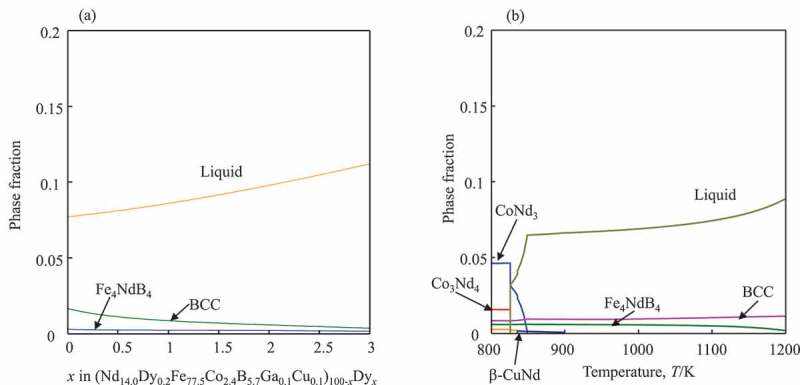
Changes of phase fractions in equilibrium with the Nd_2_Fe_14_B phase (a) during the Dy diffusion process at 1173 K, and (b) during cooling of the (Nd_14.0_Dy_0.2_Fe_77.5_Co_2.4_B_5.7_Ga_0.1_Cu_0.1_)_100-*x*_Dy*_x_* alloy with *x* = 0.6 from 1173 K

The thermodynamic calculations were performed using the constructed database on PANDAT2019 (a thermodynamic calculation software package [96]. Compared with our previous paper [95,97], where the system (Nd-Fe-B-Cu-Pr-Dy-Co-Ga) had to be simplified to the Nd-Fe-B-Cu quaternary, the coverage of the present database is closer to the real alloy systems. Since Pr is not included in the database, the alloy composition was reduced to Nd_14.0_Dy_0.2_Fe_77.5_Co_2.4_B_5.7_Ga_0.1_Cu_0.1_ (at%). The phase fractions at 1173 K as a function of Dy concentration are presented in Figure 5(a) for the alloy of (Nd_14.0_Dy_0.2_Fe_77.5_Co_2.4_B_5.7_Ga_0.1_Cu_0.1_)_100−*x*_Dy*_x_*. As the total amount of lanthanides was increased from 14.2% to 14.7% in experiments [95], the areal fraction of the Nd-rich phases in the central area, 5–6%, increased to 8–11% in the surface area of the specimen. In the calculation, the amount of liquid increased from 7.7% (*x* = 0) to 8.2% (*x* = 0.6). When the (Nd_14.0_Dy_0.2_Fe_77.5_Co_2.4_B_5.7_Ga_0.1_Cu_0.1_)_100−*x*_Dy*_x_* alloy with *x* = 0.6 was cooled to 823 K, total amount of grain boundary phases was approximately 8% (Figure 5(b)). In the calculations at low temperatures, compounds with Co become stable, which are not observed in the microstructure of magnets. One of the reasons for this discrepancy might be that partitioning of Co into the Nd_2_Fe_14_B phase is not yet included in this database. This is one of the important issues for the future update of the database. In addition to the phase fractions, the concentration of Dy in the Nd_2_Fe_14_B can also be calculated. At 1223 K it is 3.2 at%Dy, which is in good agreement with the experimentally observed value, 3.2 ~ 3.3 at%, in the Dy-containing alloy [98]. For further refinement of the present database, it demands experimental data such as fractions, constitutions and identification of phases before and after GBDPs.

For the GBDP, various rare-earth elements and its eutectic alloys such as Tb [99], Tb-Dy [100], Pr-Cu [101], Nd-Cu [102], and Nd-Ga-Cu [103] have been tried as diffusing agents. Although the present database does not cover the systems with Tb or Pr, the equilibria in some systems such as Nd-Cu can be estimated as demonstrated for the GBDP with Dy. It may be worth noting that for the eutectic GBDP one of the key factors is the selection of the lowest eutectic composition, which can be estimated from thermodynamic calculations with the use of the constructed database.

### 4.2 Hydrogenation decomposition desorption recombination (HDDR) process

The HDDR process has been widely used to make fine powders of the Nd_2_Fe_14_B phase with micrometer-sized grains [16]. This process consists in two stages. The first stage is the disproportionation stage where alloys are treated at 1000–1200 K under an H_2_ gas atmosphere to form NdH_2_. Owing to volume expansion in this hydride formation, in the first stage, fine hydride powders and their reaction products are obtained. The second stage is the recombination stage. After the first stage is completed, H_2_ gas is evacuated. As the hydride is dehydrogenated, the recombination process proceeds under vacuum, and a fine powder of Nd_2_Fe_14_B is obtained. The reactions in the HDDR process can be summarized as

(12) $$NdH_{2}^{\;}+6Fe+\frac{1}{2}Fe_{2}B\;↔\;\;\frac{1}{2}Nd_{2}Fe_{14}B+H_{2}^{\;}.$$

During the HDDR process, the phase equilibria are estimated from the present database by adding the Gibbs energies of NdH_2_ [104] and the gas phases [105]. Although the Nd-H binary system has been critically assessed by Luo et al. [106] where the NdH_2_ phase was modeled as a non-stoichiometric compound using a three sublattice model, in the present calculations the NdH_2_ phase is introduced as a stoichiometric compound for simplicity. The Gibbs energy in Eq. (3) fitted to experimental data [107–112] is thus given by

(13) $$\;G_{m}^{Nd_{1/3}H_{2/3}}=-70433+47.867T+\frac{1}{3}{\;}^{0}G_{Nd}^{DHCP}+\frac{1}{3}{\;}^{0}G_{H_{2}}^{Gas}.$$

**Figure 6.**
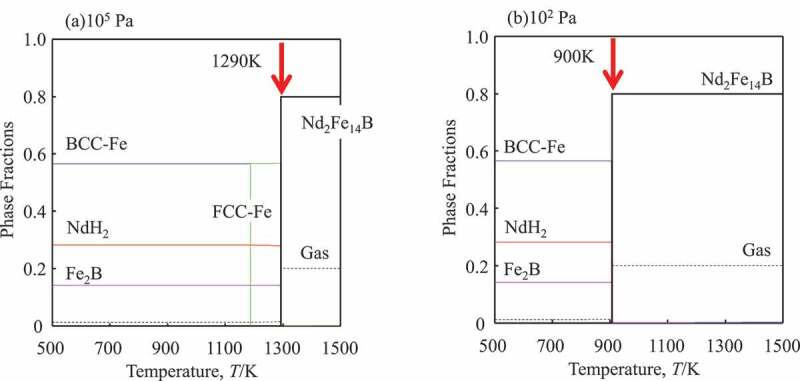
Calculated phase fractions as a function of temperature at a pressure (a) 10^5^ Pa and (b) 10^2^ Pa for the HDDR process of the Nd-Fe-B ternary alloy. Red arrows indicate the reaction temperatures

**Figure 7.**
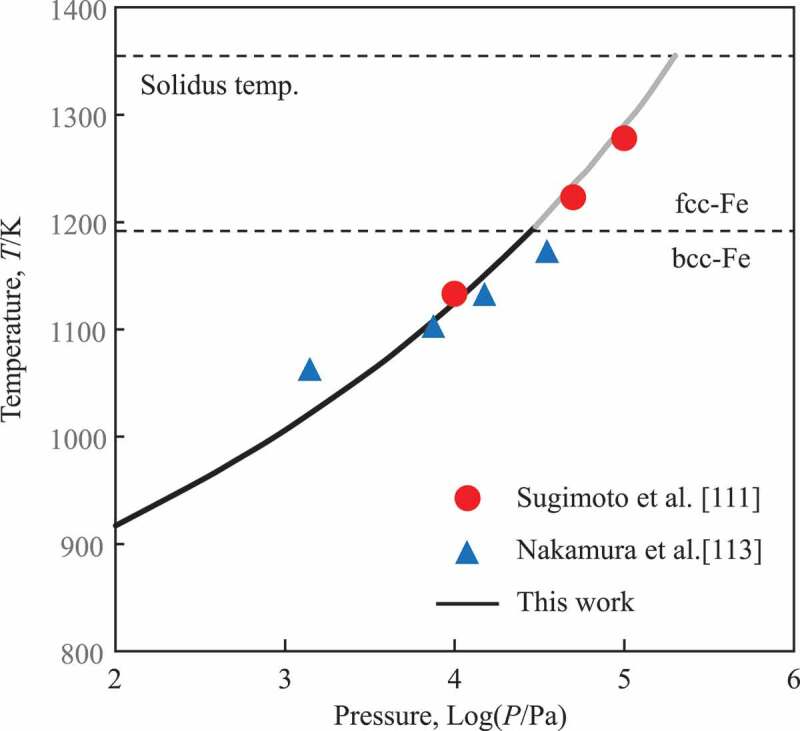
Calculated reaction temperature for Eq. (12) in the HDDR process; plots are experimental data [110,112] and the solid line is the present calculation

Although other hydrides are not included in the present version of the database, it can be applied for the fundamental system, which consists of Nd-Fe-B-H in Eq. (12). The calculated phase equilibria in the (Nd_2_Fe_14_B_1_)_80_(H_2_)_10_ (in at%) are presented in Figure 6(a,b) where NdH_2_ decomposes at 1300 K (10^5^Pa), and 900 K (10^2^Pa). Since there is an allotropic transformation of Fe at 1185 K, Fe in Eq. (12) is bcc at 10^2^Pa and fcc at 10^5^Pa. The calculated curve in Figure 7 is in good agreement with experimental results for Nd-Fe-B magnets [110,112]. This result suggests that the present database can reproduce the reaction and be applied for optimizing parameters in the HDDR processes. For further expansions and improvements to model the reactions in the alloys with Dy, it should be considered Dy-hydrides, non-stoichiometry of these compounds, and the solubility of hydrogen in solid solution phases [113].

## 5. Summary

For the Nd-Fe-B permanent magnets, thermodynamic and phase diagram information was overviewed. Based on the literature data and the present results, a prototype thermodynamic database was constructed where the Gibbs energy of the Nd_2_Fe_14_B compound and the Dy-Nd binary system were reassessed. This database covers common elements used in Nd-Fe-B magnets, i.e. Nd, Fe, B, Al, Co, Cu, Dy, and Ga. The results obtained are as follows:

(1) The magnetic excess Gibbs energy of Nd_2_Fe_14_B compound was reassessed using thoroughly measured heat capacity data. The substitution of Dy into Nd_2_Fe_14_B was described by the four sublattice model where the parameters in the Gibbs energy function were estimated from *ab initio* calculations. The Dy-Nd binary system was reassessed based on formation energies from *ab initio* calculations. The interaction parameters between Dy and Nd atoms expected from the theoretical calculations suggested that solution phases in this system might behave as ideal solutions.

(2) The constructed database was applied for actual fabrication processes of the Nd-Fe-B permanent magnets, which are the GBDP and the HDDR process. The phase equilibria in GBDP with Dy were estimated where the Dy concentrations in the Nd_2_Fe_14_B compound. By introducing the Gibbs energy of NdH_2_ and Gas phases, the database was applied for the HDDR process. It is demonstrated that the reactions in the hydrogenation and the recombination processes can be reproduced.

## Bibgraphical-note

Taichi ABE is a Dr. eng., and a senior researcher of National Institute for Materials Science since 1992.

## References

[1] Sagawa M, Fujimura S, Togawa N, et al. New material for permanent magnets on a base of Nd and Fe (invited). J Appl Phys. 1984;55(6):2083–2087. .

[2] Sagawa M. Review of sintering process development for Nd-Fe-B. J Mater Eng. 1991;13(2):95–101.

[3] Gutfleisch O, Willard MA, Brück E, et al. Magnetic materials and devices for the 21st century: stronger, lighter, and more energy efficient. Adv Mater. 2011;23(7):821–842. 21294168 10.1002/adma.201002180

[4] Sugimoto S. Current status and recent topics of rare earth permanent magnets. J Phys D: Appl Phys. 2011;44(6):064001.

[5] Nakamura H. The current and future status of rare earth permanent magnets. Scripta Mater. 2018;154:273–276.

[6] Hirosawa S. Permanent magnets beyond Nd-Dy-Fe-B. IEEE Trans Magn. 2019;55(2):2100506.

[7] Coey JMD. Perspective and prospects for rare earth permanent magnets. Engineering. 2020;6(2):119–131.

[8] Hirosawa S, Nishino M, Miyashita S. Perspectives for high-performance permanent magnets: applications, coercivity, and new materials. Adv Sci Nanosci Nanotechol. 2017;8(1):013002.

[9] Sasaki TT, Ohkubo T, Hono K. Structure and chemical compositions of the grain boundary phase in Nd-Fe-B sintered magnets. Acta Mater. 2016;115:269–277.

[10] Sasaki TT, Takada Y, Okazaki H, et al. Role of Ga on the high coercivity of Nd-rich Ga-doped Nd-Fe-B sintered magnet. J Alloys Compd. 2019;790:750–759.

[11] Matsuura Y, Hirosawa S, Yamamoto H, et al. Phase diagram of the Nd-Fe-B ternary system. Jpn J Appl Phys. 1985;24(8A):L635–L637.

[12] Tsai DS, Chin TS, Hsu SE. The phase diagrams of the psuedobinary Nd-(Fe14)B and the Fe-Nd-B ternary system. IEEE Trans Magn. 1987;23(5):3607–3609.

[13] Schneider G, H EH, Petzow G, et al. Phase relations in the system Fe-Nd-B. Z Metallkd. 1986;77:755–761.

[14] Lee RW. Hot-pressed neodymium-iron-boron magnets. Appl Phys Lett. 1985;46(8):790–791.

[15] Lee RW, Brewer EG, Schffel NA. Processing of neodymium-iron-boron melt-spun ribbons to fully dense magnets. IEEE Trans Magn. 1985;21(5):1958–1963.

[16] Nakayama R, Takeshita T, Itakura, et al. Microstructures and crystallographic orientation of crystalline grains in anisotropic Nd-Fe-Co-B-(Ga or Zr) magnet powders produced by the hydrogenation-decomposition-desorption-recombination process. J Appl Phys. 1994;76(1):412–417.

[17] Bernardi J, Fidler J, Sagawa M, et al. Microstructural analysis of strip cast Nd-Fe-B alloys for high (BH)max magnets. J Appl Phys. 1998;83(11):6396–6398.

[18] Umeda T, Okane T, Kurz W. Phase selection during solidification of peritectic alloys. Acta Mater. 1996;44(10):4209–4216.

[19] Sepehri-Amin H, Ohkubo T, Shima T, et al. Grain boundary and interface chemistry of an Nd-Fe-B-based sintered magnet. Acta Mater. 2012;60(3):819–830.

[20] Fukazawa T, Hirosawa S, Hono K. The effect of oxygen on the surface coercivity of Nd-coated Nd-Fe-B sintered magnets. J Appl Phys. 2009;105(7):07A724.

[21] Matsuura M, Goto R, Tezuka N, et al. Influence of Nd oxide phase on the coercivity of Nd-Fe-B thin films. Mater Trans. 2010;51:1901–1904.

[22] Hirota K, Nakamura H, Minowa T, et al. Coercivity enhancement by the grain boundary diffusion process to Nd-Fe-B sintered magnets. IEEE Trans Magn. 2006;42(10):2909–2911. .

[23] Akiya T, Liu J, Sepehri-Amin H, et al. High-coercivity hot-deformed Nd-Fe-B permanent magnets processed by Nd-Cu eutectic diffusion under expansion constraint. Scripta Mater. 2014;81:48–51.

[24] Lukas HL, Fries SG, Sundman B. Computational Thermodynamics, The CALPHAD method. Cambridge (UK): Cambridge University Press; 2007.

[25] Saunders N,P, Miodownik A. CALPHAD (Calculation of phase diagrams): a comprehensive guide. UK: Elsevier; 1998.

[26] Abe T, Chen Y, Saengdeejing A, et al. Computational phase diagrams for the Nd-based magnets based on the combined ab initio/CALPHAD approach. Scripta Mater. 2018;154:305–310.

[27] Hillert M, Staffansson LI. The regular solution model for stoichiometric phases and ionic melts. Acta Chem Scand. 1970;24(10):3618–3626.

[28] Sundman B. Modification of the two-sublattice model for liquids. CALPHAD. 1991;15(2):109–119.

[29] Hansen M. Constitution of binary alloys. 2nd edition ed. New York: McGraw-Hill; 1958.

[30] ASM alloy phase diagram database [Internet]. cited 2020 Oct 14; Available from: https://www.asminternational.org/materials-resources/online-databases/

[31] Gschneidner KA Jr. Rare earth alloys: acritical review of the alloy systems of the rare earth scandium and yttrium metals. Princeton, NJ: Van Nostrand; 1961.

[32] SGTE Unary database version 5.0 [Internet]. cited 2020 Oct 14; Available from: http://www.crct.polymtl.ca/sgte/

[33] Dinsdale AT. SGTE data for pure elements. CALPHAD. 1991;15(4):317–425.

[34] Redlich O, Kister AT. On the thermodynamics of solutions. VII. Critical properties of mixtures. J Chem Phys. 1962;36(8):2002–2009.

[35] Inden G. The role of magnetism in the calculation of phase diagrams. Physica B+C. 1981;103(1):82–103.

[36] Inden G. Determination of chemical and magnetic interchange energies in BCC alloys. Z Metallkd. 1975;66:577–583.

[37] Hillert M, Jarl M. A model for allying effects in ferromagnetic metals. CALPHAD. 1978;2(3):227–238.

[38] Vienna VASP. Ab initio simulation package [Internet]. Austria Kresse G;2004. cited 2020 Oct 14; Available from: http://www.vasp.at/

[39] Chen Y, Saengdeejing A, Matsuura M, et al. Formation of the Face-Centered Cubic (FCC)-NdOx phase at Nd/Nd-Fe-B interface: a first-principles modeling. JOM. 2014;66(7):1133–1137.

[40] Saengdeejing A, Chen Y, Matsuura M, et al. First–principles study of stability of Cu in the Nd–rich and Nd oxide phases of Nd–Fe–B permanent magnet. J Chin Chem Soc. 2016;63(6):506–512.

[41] Laks DB, Ferreira LG, Froyen S, et al. Efficient cluster expansion for substitutional systems. Phys Rev B. 1992;46(19):12587–12605. . 10.1103/physrevb.46.12587 10003179

[42] Van de Walle A, Asta M, Ceder G. The alloy theoretic automated toolkit: a user guide. CALPHAD 2002;26:539–553.

[43] Hillert M. Phase equilibria, phase diagrams and phase transformations. 2nd ed. Cambridge, UK: Cambridge University Press; 2008.

[44] Mirkovic D, Grobner J, Schmid-Fetzer R, et al. Experimental study and thermodynamic re-assessment of the Al–B system. J Alloys Compd. 2004;384(1–2):168–174.

[45] Ohtani H, Chen Y, Hasebe M. Phase separation of the B2 structure accompanied by an ordering in Co-Al and Ni-Al binary systems. Mater Trans. 2004;45(5):1489–1498.

[46] Minic D, Premovic M, Cosovic V, et al. Experimental investigation and thermodynamic calculations of the Al–Cu–Sb phase diagram. J Alloys Compd. 2013;555:347–356.

[47] Cacciamani G, De Negri S, Saccone A, et al. The Al-R-Mg (R = Gd, Dy, Ho) Systems. Part II: thermodynamic modeling of the binary and ternary systems. Intermetallics. 2003;11(11–12):1135–1151.

[48] Ansara I, Dinsdale AT, Rand MH. COST 507: thermochemical database for light metal alloys volume 2. Luxembourg: Office for Official Publications of the European Communities; 1998.

[49] Watson A. Re-assessment of phase diagram and thermodynamic properties of the Al-Ga system. CALPHAD. 1992;16(2):207–217.

[50] Gao MC, Unlu N, Shiflet GJ, et al. Reassessment of Al-Ce and Al-Nd binary systems supported by critical experiments and first-principles energy calculations. Metal Mater Trans A. 2005;36A:3269–3279.

[51] Du Y, Schuster JC, Chang YA, et al. Thermodynamic description of the B-Co system: modeling and experiment. Z Metallkd. 2002;93(11):1157–1163.

[52] Wang CP, Guo SH, Tang AT, et al. Thermodynamic assessments of the Cu-B and Cu-Tm systems. J Alloy Compd. 2009;482(1–2):67–72.

[53] Li S, Rong MH, Xu L, et al. Thermodynamic assessment of the RE-B (RE=Cr, Dy, Lu) binary systems. 2020(68):101704.

[54] Hallemans B, Roos JR. Thermodynamic reassessment and calculation of the Fe-B phase diagram. Z Metallkd. 1994;85(10):676–682.

[55] Li X, Cheng K, Yuan X, et al. Thermodynamic assessment of the Ga-X (X=B, Ca, Sr, Ba) systems supported by first-principles calculations. CALPHAD 2013;43:52–60.

[56] Hallemans B, Wollants P, Roos JR. Thermodynamic assessment of the Fe-Nd-B phase diagram. J Phase Equilib. 1995;16(2):137–149.

[57] Palumbo M, Curiotto S, Battezzati L. Thermodynamic analysis of the stable and metastable Co-Cu and Co-Cu-Fe phase diagrams. CALPHAD. 2006;30(2):171–178.

[58] Su X, Zhang W, Du Z. Thermodynamic Modeling of the Co-Dy system. Z Metallkd. 1998;89(8):522–526.

[59] Ohnuma I, Enoki H, Ikeda O, et al. Phase Equilibria in the Fe-Co binary system. Acta Mater. 2002;50(2):379–393.

[60] Chari A, Garay A, Arroyave R. Thermodynamic remodeling of the Co-Ga system. CALPHAD. 2010;342(2):189–195.

[61] Liu X, Du Z, Guo C, et al. Thermodynamic assessment of the Co-Nd system. J Alloys Compd. 2007;439(1–2):97–102. .

[62] Zhang LG, Huang GX, Qi HY, et al. Thermodynamic assessment of the Cu-Dy binary system. J Alloys Compd. 2009;470(1–2):214–217. .

[63] Turchanin MA, Agraval PG, Nikolaenko IV. Thermodynamics of alloys and phase equilibria in the copper-iron system. J Phase Equilib. 2003;24(4):307.

[64] Li J-B, Ji LN, Liang JK, et al. A thermodynamic assessment of the copper-gallium system. CALPHAD. 2008;32(2):447–453. .

[65] Wang P, Zhou L, Du Y, et al. Thermodynamic optimization of the Cu-Nd system. J Alloy Compd. 2011;509(6):2679–2683. .

[66] Landin S, Agren J. Thermodynamic assessment of Fe-Tb and Fe-Dy phase diagrams and prediction of Fe-Tb-Dy phase diagram. J Alloys Compd. 1994;207-208:449–453.

[67] Moore EE, Turchi PEA, Landa A, et al. Development of a CALPHAD thermodynamic database for Pu-U-Fe-Ga alloys. Appl Sci. 2019;9(23):5040.

[68] Okamoto H. Dy-Ga (Dysprosium-Gallium). J Phase Equilib Diff. 2008;29(2):205.

[69] Ao WQ, Li JQ, Jian YX, et al. Reinvestigation of the phase diagram of the Dy-Ga binary system. CALPHAD. 2007;31(2):233–236.

[70] Yatsenko SP, Semenov BG, Chuntonov KA. Ce-Ga, Nd-Ga and Sm-Ga phase diagrams. IzvAkad Nauk SSSR Met. 1977;6(6):185–187.

[71] Yatsenko SP, Semyannikov AA, Semenov BG, et al. Phase diagrams of rare earth metals with gallium. J Less-Common Met. 1979;64(2):185–199. .

[72] Kimmel G, Dayan D, Grill A. The Gallium-rich side of the Nd-Ga and Ce-Ga systems. J Less Common Met. 1980;75(1):133–140.

[73] Monory R, Pelleg J, Grill A. The Neodymium-Gallium system. J Less-Common Met. 1978;61(2):293–299.

[74] Cirafici S, Franceschi E. Stacking of close-packed AB_3_ layers in RGa_3_ compounds (R=heavy rare earth). J Less-Common Met. 1981;77(2):269–280.

[75] Pelleg J, Zevin I, Kimmel G, et al. Recent advances in the rare earth-gallium systems (R-Ga). J Less-Common Met. 1985;110(1–2):91–97. .

[76] Liu YQ, Zhao XS, Yang J, et al. Thermodynamic optimization of the boron–cobalt–iron system. J Alloy Compd. 2011;509(14):4805–4810. .

[77] Zhang LG, Chen XM, Dong HQ, et al. Thermodynamic assessment of Al–Cu–Dy system. J Alloys Compd. 2009;480(2):403–408.

[78] Chen HL, Du Y, Xu H, et al. Experimental investigation and thermodynamic modeling of the ternary Al-Cu-Fe system. J Mater Res. 2009;24(10):3154–3164.

[79] Turchanin MA, Dreval LA, Abdulov AR, et al. Mixing enthalpies of liquid alloys and thermodynamic assessment of the Cu–Fe–Co system. Powder Metall Metal Ceram. 2011;50(1–2):98–116.

[80] Saeki M, Horino Y, Jinya L, et al. Thermodynamic analysis of phase Equilibria in the Nd-Fe-Cu ternary system. J Japan Inst Met Mater. 2017;81(1):32–42.

[81] Computational phase diagram database (CPDDB)[Internet]. cited 2021 Apr 12; Available from: https://cpddb.nims.go.jp/en

[82] Van Ende MA, Jung IH. Critical thermodynamic evaluation and optimization of the Fe-B, Fe-Nd, B-Nd and Nd-Fe-B systems. J Alloys Compd. 2013;548:133–154.

[83] Van Ende MA, Jung IH, Kim YH, et al. Thermodynamic optimization of the Dy-Nd-Fe-B system and application in the recovery and recycling of rare earth metals from NdFeB magnet. Green Chem. 2015;17(4):2246–2262. .

[84] Zhou GJ, Luo Y, Zhou Y. Thermodynamic reassessment of the Nd-Fe-B ternary system. J Electronic Mater. 2015;45(1):418–425.

[85] Chen TL, Wang J, Guo CP, et al. Thermodynamics description of the Nd-Fe-B ternary system. CALPHAD 2019;66:101627.

[86] Hanindriyo AT, Sridar S, Hari Kumar KC, et al. Ab initio thermodynamic properties of certain compounds in Nd-Fe-B system. CALPHAD 2020;180:109696.

[87] Morishita M, Abe T, Nozaki A, et al. Calorimetric study of Nd_2_Fe_14_B: heat capacity, standard Gibbs energy of formation and magnetic entropy. Thermochim Acta. 2020;690:178672.

[88] Luis F, Mate B, Pique C, et al. A thermodynamic study of the R2Fe14X; X=B,C at the Curie temperature. J Mag Mag Mater. 1991;101:414–416.

[89] Saito K, Doi S, Abe T, et al. Quantitative evaluation of site preference in Dy-substituted Nd2Fe14B. J Alloys Compd. 2017;721:476–481.

[90] Arajs S, Colvin RV, Chessin H. Electrical and X-ray studies of some neodymium-dysprosium alloys. J Less-Common Met. 1965;8(3):186–194.

[91] Chatterjee D, Taylor KNR. Magnetic and structural properties of the neodymium-dysprosium alloy system. J Phys F. 1972;2(1):151–158.

[92] Kobzenko GF, Svechnikov VN, Matrynchuk EL. Phase diagram of the system neodymium-dysprosium. Dopov Akad Nauk Ukr RSR Ser A. 1972;6:563–565.

[93] Gschneidner KA, Calderwood FW. The Dy-Nd (Dysprosium-Neodymium) system. Bull Alloy Phase Diag. 1982;3(3):348–350.

[94] Gschneidner KA, Calderwood FW, editors. Gschneidner KA, Eyring L. Intra rare earth binary alloys: Phase relationships, lattice parameters and systematics. Handbook on the physics and chemistry of rare earths. Amsterdam, North-holland physics publishing; 1982:1–162.

[95] Seelam UMR, Ohkubo T, Abe T, et al. Faceted shell structure in grain boundary diffusion-processed sintered Nd-Fe-B magnets. J Alloys Compd. 2014;617:884–892.

[96] Chen SL, Daniel S, Zhang F, et al. The PANDAT software package and its applications. CALPHAD 2002;26:175–188.

[97] Sepehri-Amin H, Ohkubo T, Hono K. The mechanism of coercivity enhancement by the grain boundary diffusion process of Nd-Fe-B sintered magnets. Acta Mater. 2013;61(6):1982–1990.

[98] Kim TH, Sasaki TT, Ohkubo T, et al. Microstructure and coercivity of grain boundary diffusion processed Dy-free and Dy-containing Nd-Fe-B sintered magnets. Acta Mater. 2019;172:139–149.

[99] Kim TH, Sasaki TT, Koyama T, et al. Formation mechanism of Tb-rich shell in grain boundary diffusion processed Nd-Fe-B sintered magnets. Scripta Mater. 2020;178:433–437.

[100] Li W, Zhang Q, Zhu Q, et al. Formation of anti-shell/core structure of heavy rare earth elements (Tb, Dy) in sintered Nd-Fe-B magnet after grain boundary diffusion process. Scripta Mater. 2019;163:40–43.

[101] Sepehri-Amin H, Liu L, Ohkubo T, et al. Microstructure and temperature dependent of coercivity of hot-deformed Nd-Fe-B magnets diffusion processed with Pr-Cu alloy. Acta Mater. 2015;99:297–306.

[102] Sepehri-Amin H, Ohkukbo T, Nagashima S, et al. High-coercivity ultrafine-grained anisotropic Nd-Fe-B magnets processed by hot deformation and the Nd-Cu grain boundary diffusion process. Acta Mater. 2013;61(17):6622–6634.

[103] Liu L, Sepehri-Amin H, Sasaki TT, et al. Coercivity enhancement of Nd-Fe-B hotdeformed magnets by the eutectic grain boundary diffusion process using Nd-Ga-Cu and Nd-Fe-Ga-Cu alloys. AIP Adv. 2018;8(5):056205.

[104] Korst WL, Warf JC. Rare earth-hydrogen systems. I. Structural and thermodynamic properties. Inorg Chem. 1966;5(10):1719–1726.

[105] SGTE substance database[Internet]. cited 2020 Oct 14; Available from: https://www.sgte.net/en/neu

[106] Luo Q, Chen SL, Zhang JY, et al. Experimental investigation and thermodynamic assessment of the Nd-H and Nd-Ni-H systems. CALPHAD 2015;51:282–291.

[107] Peterson DT, Poskie TJ, Straatmann JA. Neodymium-Neodymium hydride phase system. J Less-Common Met. 1971;23(2):177–183.

[108] Mulford RNR, Holley CE. Pressure-temperature-composition studies of some rare earth/hydrogen systems. J Phys Chem. 1955;59(12):1222–1226.

[109] Book D, Harris IR. Hydrogen absorption/desorption and HDDR studies on Nd16Fe76B8 and Nd11.8Fe82.3B5.9. J Alloy Compd. 1995;221(1–2):187–192.

[110] Sugimoto S, Gutfleisch O, Harris IR. Resistivity measurements on hydrogenation disproportionation desorption recombination phenomena in Nd-Fe-B alloys with Co, Ga, and Zr additions. J Alloys Compd. 1997;260(1–2):284–291.

[111] Bonnet JE, Daou JN. Rare-earth dihydride compounds: lattice thermal expansion and investigation of the thermal dissociation. J Appl Phys. 1977;48(3):964–968.

[112] Nakamura H, Kato K, Book D, et al. A thermodynamic study of the HDDR conditions necessary for anisotropic Nd-Fe-B powders. Proceedings of the 15th International Workshop on Rare Earth Magnets and Their Applications; 1998 Jul 1; Dresden, Germany: TIB Hannover; p. 507–516.

[113] Fu K, Li G, Li J, et al. Experimental study and thermodynamic assessment of the dysprosium-hydrogen binary system. J Alloy Compd. 2017;696:60–66.

